# Structural and Functional Insights into the Transmembrane Domain Association of Eph Receptors

**DOI:** 10.3390/ijms22168593

**Published:** 2021-08-10

**Authors:** Amita R. Sahoo, Matthias Buck

**Affiliations:** 1 Department of Physiology and Biophysics, School of Medicine, Case Western Reserve University, 10900 Euclid Avenue, Cleveland, OH 44106, USA; amita.sahoo@case.edu; 2 Department of Neurosciences, School of Medicine, Case Western Reserve University, 10900 Euclid Avenue, Cleveland, OH 44106, USA; 3 Department of Pharmacology, School of Medicine, Case Western Reserve University, 10900 Euclid Avenue, Cleveland, OH 44106, USA; 4 Case Comprehensive Cancer Center, School of Medicine, Case Western Reserve University, 10900 Euclid Avenue, Cleveland, OH 44106, USA

**Keywords:** receptor tyrosine kinase (RTKs), Eph receptors, TM dimerization, transmembrane domain (TMD)

## Abstract

Eph receptors are the largest family of receptor tyrosine kinases and by interactions with ephrin ligands mediate a myriad of processes from embryonic development to adult tissue homeostasis. The interaction of Eph receptors, especially at their transmembrane (TM) domains is key to understanding their mechanism of signal transduction across cellular membranes. We review the structural and functional aspects of EphA1/A2 association and the techniques used to investigate their TM domains: NMR, molecular modelling/dynamics simulations and fluorescence. We also introduce transmembrane peptides, which can be used to alter Eph receptor signaling and we provide a perspective for future studies.

## 1. The Family of Eph Receptors, Their Domain Structure and Function

Erythropoietin-producing hepatocellular carcinoma receptors (Ephs) represent the largest superfamily of tyrosine kinases receptors (RTKs). Ephs are expressed in most tissues during embryogenesis and are essential for a large variety of developmental processes. [1] They play major roles in axon guidance, development and organogenesis [2], but some can also be dysregulated and become drivers for cancers [3,4,5,6]. Out of 16 Ephs that have been identified in animals, 14 are known to express in humans. Based on their sequence homology and how they interact with the membrane-anchored ephrin ligands, these Eph receptors are divided into two subclasses. EphA, consisting of nine receptors (EphA1-A8 and EphA10), and EphB, consisting of five receptors (EphB1-B4 and EphB6). Eph receptors are activated by interacting with membrane-anchored ligands, the ephrins and are thus different from other RTKs which are activated by soluble ligands. These ephrins are also divided into two subclasses: A-type ephrins (A1-A6) and B-type ephrins (B1-B3) [7]. EphA binds preferentially to A-type ephrins and EphB to B-type ephrins. However, the exception is EphA4 which interacts with both A- and B-type ephrins and other cross-interactions, also between different Eph receptors likely exist [8].

**Figure 1:**
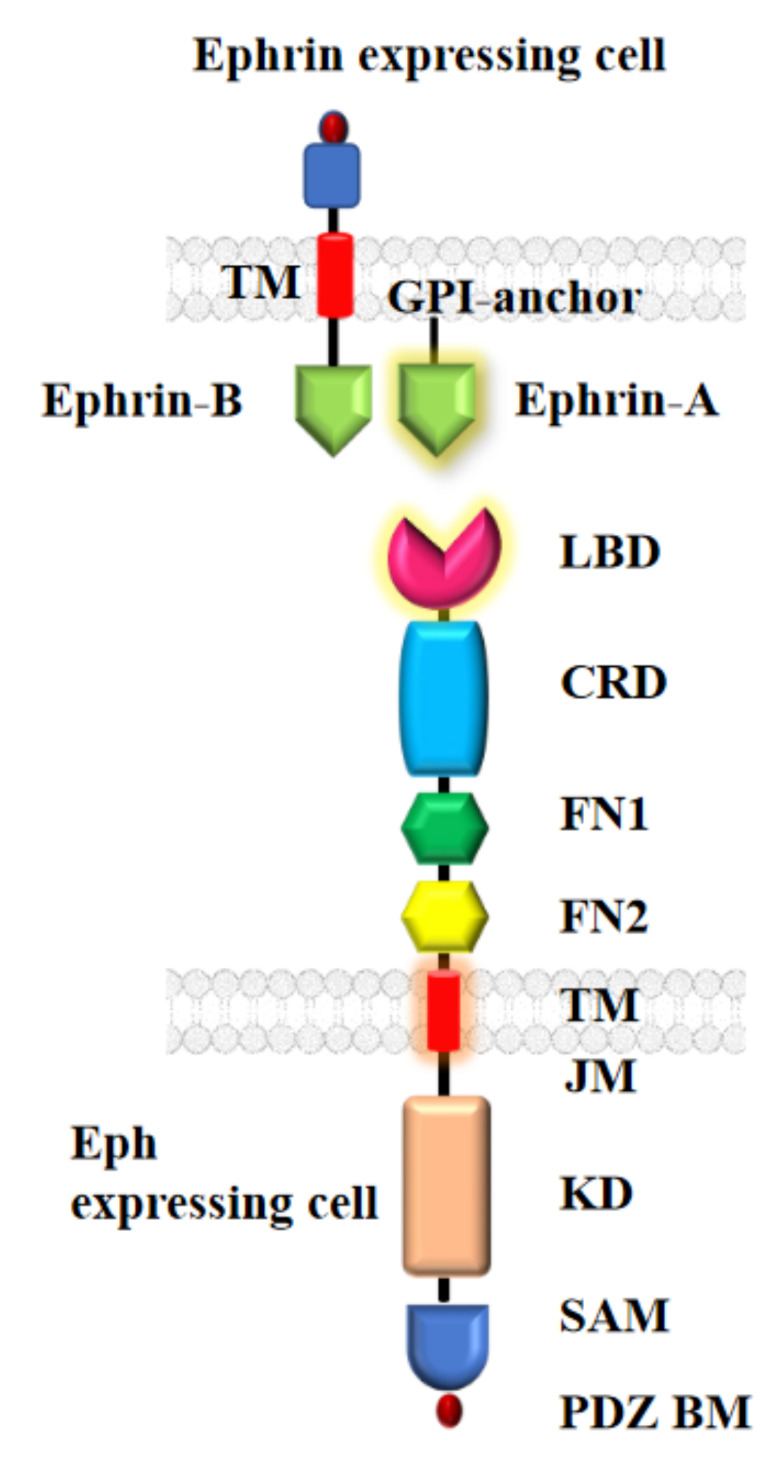
Structural overview of Eph-ephrin complex. An Eph receptor has three distinct regions: Extracellular region consisting of ligand binding domain (LBD), cysteine-rich domain (CRD) and two fibronectin-III like domains (FN1 and FN2); Transmembrane domain (TMD); and Intracellular region consisting of juxta membrane region (JM), kinase domain (KD), sterile alpha motif (SAM) and a PDZ binding motif (PDZ BM). Both ephrinA (GPI-anchored) and ephrinB (transmembrane) ligands interact with the LBD of the Eph receptor.

The functional signaling unit of Ephs is usually assembled as dimers if not higher order oligomers from two or more identical or nonidentical receptors typically by the binding of the ephrin ligand. Glycosylation of Eph receptor binding domain as well as on ephrin are reported to contribute to the interactions [9,10]. The general structure of Ephs is highly conserved throughout the animal kingdom [11] and consists of three distinct regions; the extracellular region (ECR) [7,8,9,10,11], the transmembrane domain (TMD) [12], and the intracellular region (ICR) [13] (details of the domain organization are given in Figure 1 and its Legend). Generally, the ECRs represent the longest region, consisting of an *N*-terminal globular domain for ephrin binding known as the ligand-binding domain (LBD), a cysteine-rich EGF-like domain (CRD) and two fibronectin III repeats [12,13]. The LBD, together with the CRD is additionally involved in ephrin-independent receptor dimerization and clustering [14,15]. The TMDs constitute the shortest (~25 residues) region, which typically forms a helix and which connects the ECR and ICR through an unidirectional insertion into the membrane bilayer [16]. The ICR is comprised of a juxta membrane (JM) domain, a tyrosine kinase domain (KD), sterile alpha motif (SAM) and PDZ binding motif (PDZBM) [17]. Eph receptors generally function by canonical signalling through the ligand-induced clustering via tyrosine kinase activation, adaptor protein binding etc., whereas non-canonical signalling may involve smaller clusters, less tyrosine kinase activity and serine phosphorylation of a linker region between KD and SAM domains. This phosphorylated linker then interacts with adaptor proteins and appears to affect the protein kinase B, also known as AKT, and other Ser/Thr kinases. The detailed mechanism of signal transduction through the TM region of Eph receptors is only partly characterized, as discussed below. Specifically, Ephs are known to function by adopting multiple conformations through ligand binding induced stabilization but can also signal in a ligand independent manner [3]. The conformational flexibility of Ephs and other type I receptors (i.e., proteins which only cross the membrane once) near the membrane makes them notoriously difficult to characterize structurally. To date no high-resolution structure of a full length Eph has been solved.

**Figure 2:**
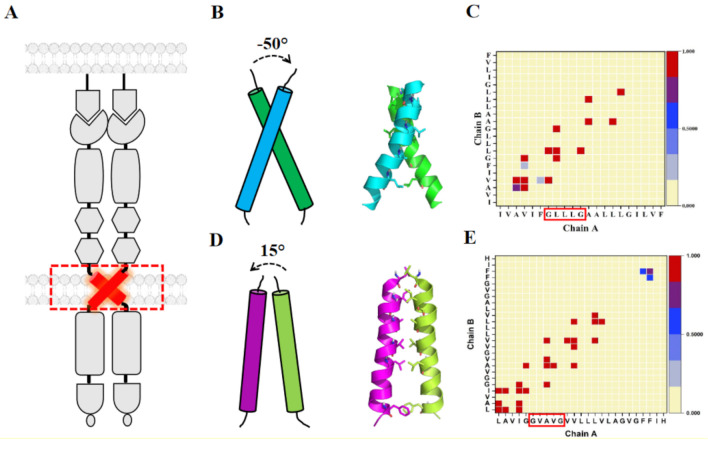
(**A**) Schematic representing lateral dimerization of Eph receptors shows the involvement of TM domains. (**B**) NMR structure of EphA1 TM dimer structure showing a right-handed configuration with an inter-helical angle of −50° and the contact map interface of EphA1 (**C**). (**D**) NMR structure of EphA2 TM dimer structure showing a left-handed configuration with an inter-helical angle of 15° and the contact map interface of EphA2 (**E**). Contact maps are calculated with a cut off 4 Å considering all the ensembles of the NMR structure. The color (white to blue to red) indicates the fractional occupation of the contact (0 to 1). GXXXG motifs are highlighted in the contact map of EphA1 and EphA2 (for chain A).

Several studies support the view that the membrane-embedded TMDs of RTKs including Ephs participate in the regulation of receptor chain associations as well as cross-membrane signal transduction by changing their configuration, i.e., the angle and surface of helix-helix contacts [18,19]. Several studies demonstrated that lateral dimerization of TMDs in RTKs is essential for signal transduction beyond the plasma membrane barrier [18,20], including for EphA1 and EphA2 helices, which self-associate in bicelles- a model system for a lipid bilayer membrane- [21,22] thereby likely forming the same homodimers (shown in Figure 2A) in the cell membrane [23,24,25]. Although not yet reported for Eph receptors, single point mutations in many other RTK TMDs are known to modulate the efficiency and stability of dimerization states which can thus lead to dramatic changes in the biological function of the receptors [26]. Therefore, understanding the interactions involved in TM–TM recognition inside the membrane is an important challenge. Moreover, recent studies have demonstrated how the activity of specific Ephs may be controlled by small peptides that specifically recognize their TMDs, and thereby change how the TM helices bind to one another (their configuration) [27,28]. Thus, the conserved role of TMDs in regulating the function of Eph receptors makes them promising targets for therapeutic intervention. We here provide an overview of recent studies of the structural landscape of the TM domain of Eph receptors which used a repertoire of different techniques. We discuss the progress and challenges of these structural studies and consider the integration of TMD studies with those of the ECR and ICR regions. Finally, we comment on the prospect of obtaining a medium to high resolution structure of the whole length receptor.

## 2. Available Structural Information for Eph Receptor TMDs: Use of Solution NMR

The traditional workhorse of structural biology, x-ray crystallography, has generally experienced problems with the crystallization of single-pass membrane proteins and only relatively recently have TMD structures been obtained using crystallization in the cubic lipid phase [29], which however can lead to non-physiological structures. Cryo-electron microscopy is able to resolve near atom resolution structures for extra- or intracellular domains, but at present, it seems the TMD introduces too much flexibility [30,31]. No structures for a natural full-length Eph receptor embedded in a lipid bilayer or mimetic have been reported to date. Solution NMR is challenged with sample preparation as well as the large molecular weight of the full-length proteins, but studies of the TMD have been successful for helix dimer structures of EphA1 and −A2 [21,22]. In principle solution NMR is the method of choice to deal with conformational and configurational dynamics, that is motions within a protein and motions of domains relative to one another [32,33]. However, the motions should be either fast (ps-ns) or slow (ms and lesser frequency of transitions) as in the so-called intermediate exchange regime, signals broaden and spectra become difficult, if not impossible to analyze. Thus, in some TMDs mutations were made to alter their dynamics and also the number of states populated [34]. Another important factor for NMR is the lipid, if not detergent used to model the properties of the lipid bilayer of the cellular plasma membrane. In some cases, only dodecylphosphocholine (DPC) or other detergents were found suitable. But detergents can bind to the hydrophobic residues in any orientation, typically forming a globule around the TMD [35]. Bicelles by contrast have a bilayer-like region composed of lipids such as dimyristoylphosphatidylcholine (DMPC), bound on the outside by dihexanoylphosphatidylcholine (DHPC) detergent. These discs have a diameter of 100 Å and have seen wide ranging use [36]. However, for increased stability of such membrane models, the use of nanodiscs and polymer bound discs (e.g., SMALPS) have recently become popular [35,37]. There are only very few studies of single pass receptor TMDs yet, and neither membrane model has been used for Eph receptors.

Several areas should be mentioned which are challenging for NMR solution structure determination: For the determination of homodimer structures, typically both peptides are isotopically labeled and in such a case the hydrogen nuclei in the helices are equivalent in terms of transferring NMR signals between them (the nuclear Overhauser effect or NOE), leading to symmetric distance restraints and symmetric structures. However, NMR, as well as simulations and modelling, suggest that helices can slide relative to one another which may populate non-symmetric structures. Chemical shift perturbation is often utilized in addition to interhelix sidechain-sidechain or sidechain-mainchain distances to indicate the area of contact, but also the extent of helicity (suggested by the extent of chemical shift perturbation which arises compared to α-helices in soluble proteins). Often helical restraints are put into the structure calculation as ideal alpha-helix backbone restraints, which would “iron out” most helix bending or kinking. Chemical shifts may also be perturbed due to longer range allosteric effects and might not reliably indicate the area of closest contact. Instead, it has been suggested that the strongest contacts are indicated by a rigidification of sidechain dynamics [38].

The solution NMR structure of the TMD homodimer of EphA1 was obtained in lipid bicelles under two different conditions: at pH 4.3 (PDB ID: 2K1K) and pH 6.3 (PDB ID: 2K1L). The EphA1 sequence is unique by comparison to the other Eph family receptors because of the presence of a membrane-embedded ionogenic residue (Glu547) at the *N*-terminus of the TMD. Under acidic conditions, the *N*-terminus of the transmembrane helix is stabilized by the carboxyl group of Glu547, whereas its deprotonation results in a fractional unfolding of the helix, and rearrangement of hydrogen bonds and of helix-helix packing [22]. This indicates that local perturbations such as pH changes and membrane lipid composition could alter TMD structural dynamics and hence, could regulate EphA1 conformational flexibility and activation. Indeed, the NMR structure of a low pH configuration was determined, where the neutral Glu547 forms an additional H-bond and helical turn, then utilizing a *C*-terminal Gly-X3-Gly motif (see below), compared to the structure at higher pH, shown in Figure 2. The latter is a right-handed crossing dimer, utilizing a more—it seems family conserved— *N*-terminal motif, see discussion below [PDB ID: 2K1L].

Identification of several characteristic dimerization motifs in the TM region sequences of Eph receptors indicates that TMD associations in the plasma membrane are dynamic and independent of ligand-induced dimerization/clustering events. The NMR structure of the dimeric TMD of EphA2 was also obtained in lipid bicelles at pH 5 (PDB ID: 2K9Y) [21]. Compared with the right-handed TM dimer of EphA1, the EphA2 TM dimer shows the left-handed arrangement of TM helices embedded into lipid bicelles, evidence that TM domains of the Eph receptors can self-associate in different configurations. This points to the diversity in the formation of TMDs within a family of RTKs and hence, is evidence for the rotation coupled mechanism of activation of these receptors [39,40]. This mechanism posits that the kinase domains are relatively rigidly attached to the TMD and information from the outside of the cell, specifically ligand binding is transmitted as a mechanical event through the membrane. In one “inactive” state kinase domains face away from each other, whereas rotation of helices by 180 degrees would bring them into closer contact for cross-phosphorylation and kinase activation [39]. Thus, let us look in more detail at the dimerization motifs in the TMD.

### 2.1. Dimerization Motifs for TM Association

In general, the association of transmembrane (TM) α-helices is controlled by many factors in the cell, such as the primary structure of interacting helices, lipid composition of their local environment, binding of external or internal ligands, and general physicochemical properties of the membrane. However, as a membrane is highly dynamic environment for proteins and peptides this might locally induce or stabilize one out of several possible TM structures. As mentioned above, experimental techniques for the determination of TM helical dimer spatial structures by NMR spectroscopy in detergent micelles, or more complicated membrane mimics (bicelles) [41] typically account only for one particular conformation of a dimer promoted by such environment [42].

The most studied interaction motifs for TMDs of membrane proteins are the GAS and heptad motifs. The GAS-motif (the so-called glycine zipper motif), occurs in ~50% of all TMDs [43]. Also known as GASright-motif, the five-residue long motif (small-X3-small residues, where small residues are mostly Glycine and sometimes Alanine or Serine) that maintains the right-handed association of TM dimers. The G-X_3_-G form of this motif (here using an alternate common notation) is mostly observed to participate in oligomerization. Smaller residues in GAS motifs allow for close proximity between opposite helical backbones and hence, enable interhelical backbone or main chain hydrogen bonds [44]. However, several published references on the affinity of TMD dimers with G-X_3_-G motifs suggest that these motifs are not essential for TMD dimerization [43,45]. Also, there is no correlation in general between the presence of GAS-motifs and the measured dimerization propensity [43]. The heptad motif (also called leucine zipper/GASleft-motif) is responsible for the left-handed packing of TM dimers. This motif contains a seven residue (abcdefg) stretch, where a and d are generally nonpolar residues that form the hydrophobic core at the interhelix interface in coiled-coil structures of soluble proteins [46,47]. Along with the hydrophobic aliphatic residues, aromatic residues also enhance the association of TMDs. Moreover, because of the low diversity of amino acids in the TMD, heptad motifs can almost always be assigned regardless of any true relevance [45].

**Figure 3:**
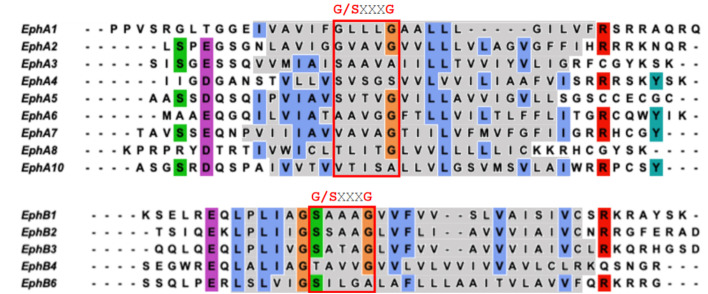
Sequence alignment of TMD of EphA and EphB receptors expressed in humans. The Small-X_3_-Small motif/Glycine zipper motifs are marked. Residues of the TM region are highlighted in grey. Conserved residues are shown in different colors.

The TM dimer of EphA1 associates with a right-handed parallel configuration [22] (Figure 2B) with helix crossing angle −50° through the *N*-terminal “glycine zipper” motif A-X_3_-G-X_3_-G, composed of residues with small side chains allowing the close approach of the helices (Figure 2B,C). MD simulation studies on the NMR structure of EphA1 TM dimer in the DMPC bilayer resulted in a stable right-handed conformer [22]. By contrast, the TM dimer of EphA2 associates with a left-handed configuration [21] (Figure 2D) with the helix crossing angle of 15° through the extended heptad repeat motif L-X_3_-G-X_2_-A-X_3_-V-X_2_-L (Figure 2D,E). Indeed, the currently resolved TMD homodimer structures of RTKs have been shown to have two different ways of packing, considering the inter-helical crossing angle; left-handed (with positive values) and right-handed (with negative values of the crossing angle). Therefore, it is suggested that both right- and left-handed variants of TM dimerization are quite common for TM helix packing of integral membrane proteins. However, the thickness and the composition of the lipid bilayer and the positioning of the juxtamembrane residues are also known to affect the configurational states of the EphA2 TMD [48]. The sequence alignment of the TMD of Eph receptors (shown in Figure 3) reveals the presence of these GAS- and small-X_3_-small residue motifs. The TMD of EphA1 and EphA2 possess the G-X_3_-G motif, whereas other EphA receptors (EphA5-EphA8) have S-X_3_-G motifs with S position is replaced by A/V/T and the G position is also occupied by A/S. Remarkably, this G-X_3_-G motif is not seen in the case of EphA10. However, the EphB receptors have a quite well conserved S/T-X_3_-G/A motif. Possibly these motifs might interact and play a significant role in TM dimerization.

### 2.2. Prediction and Computational Modelling of TM Dimers

As mentioned in the sections above, it is challenging to obtain structural and dynamic information on non-covalently bonded receptor oligomers in the membrane environment, especially if there are several states which are populated. This problem has been resolved by various strategies including theoretical and physicochemical techniques. Integration of results from several techniques is particularly helpful in suggesting the structural-dynamic details of TMD-TMD and TMD-membrane interactions at atomic, if not residue-level resolution. However, several in silico approaches have been shown to provide a reasonably quick and efficient tool for assessment of the mode of TMD association in membranes, especially when direct experimental techniques fail or are highly resource consuming. These in silico approaches can be subdivided into two major categories: ab initio molecular prediction based on sequence or packing features and molecular modelling integrated with molecular dynamics simulations.

Some prediction techniques are based on a statistical analysis of the frequency of amino acid residues and the presence of such sequence of patterns on both the helices, which form interhelical contacts. However, others, such as the PREDDIMER program [49], one of the most widely used for ab initio prediction of TM homo/heterodimeric structures of membrane proteins, is based on the alignment of the peptide’s surfaces to obtain the best complementarity of hydrophobic (molecular hydrophobicity potential, MHP). The program can be run for different pH conditions but considers the hydrophobic-membrane imbedded section of the TMD only. It delivers the predicted coordinates/pdb files of the most well packed several structures, ranked by a parameter, Fscor, which when above 2.5 generally suggests that the helices reliably dimerize. Nevertheless, generally one of the top 3–4 structures obtained, shows a close similarity with the existing NMR dimer structures for RTKs and other TMD dimers [42]. A limitation of this program is that it does not allow different membrane compositions, known to affect the charge at the membrane surface and/or thicknesses, and which in turn are known to affect at least some of the structures. The same is true for *N*- and *C*-terminal extensions, which as we saw in the case of an *N*-terminal Glu in EphA1 can have a pH-dependent effect on the structure. Of particular note in this context is the juxtamembrane region on the *C*-terminal side of the hydrophobic TM segment which typically contains a positive-charge plug, that is, several Arg and Lys which likely prevent the further translocation of the polypeptide chain into the membrane [50]. Thus, it makes sense to include a region of 6–8 amino acids at both ends of the membrane-embedded TM region in MD simulations. In some cases, an isolated even charged residue, such as Glu and Lys/Arg has been shown to “snorkel”, allowing its charged sidechain among the lipid headgroups but positioning the sidechain aliphatic tail among the lipid tails [51]. A second feature which has been examined for the whole family of RTKs using MD simulations is the tendency of the juxtamembrane region, often rich in Arg/Lys residues to interact with PIP2 (Phosphatidylinositol 4,5-bisphosphate) [50].

Molecular dynamics (MD) simulations are a key tool in structural biology to sample the conformational/configurational space of structures, while at the same time revealing the timescale of the fluctuations of the structures, as they experience dynamics in local energy minima or transition between several states [52]. In one study, several years ago we used a modelling/dynamics procedure to build an initial model for the EphA1 TMD dimer [53], in another PREDDIMER predicted best packed helix dimers were been run in all-atom (AA) MD simulations with the addition of native juxtamembrane residues to further equilibrate the model structures [54]. However, while 500 ns to a few μs are enough to relax these structures, they are not sufficient in most cases to observe a TMD-TMD dissociation in the membrane and a TMD-TMD rebinding. For this molecular modelling of TMD monomers, their insertion into the membrane and then relatively extensive molecular dynamics simulation, has become a standard procedure which allows the peptides to diffuse together [55]. However, the peptide-lipid bilayer-solvent system is large and motions of the peptide in the lipid bilayer are relatively slow (diffusion of lipids is <5Å in 50 ns at 310 K), so all-atom simulations become unfeasibly expensive computationally. The use of coarse-grained representations/potential functions reduces the number of particles several-fold but also provides a smoother energy landscape, causing a speed-up in motions by up to 100-fold.

Coarse grained (CG) simulations of glycophorin A, EphA1 and EphA2 are reported in the literature [56,57,58,59], involved the modelling of the TM region from the sequence as regular helices placed 55 Å apart in the lipid bilayer of choice. Typically, 4 µs CG simulation is sufficient in our hands to allow the TMD peptides to associate. Most of the CG simulation studies used Martini 2 force fields, which, however, has several limitations and shows excessive aggregation with very high protein-protein binding energy [60,61]. Recently, a new Martini 3 force field has been introduced [62] and it has improved the balance of all non-bonded interaction, also with the addition and re-parametrization of new beads and labels which results in more accurate prediction of protein-protein and protein-lipid interactions. A recent study also shows the great correlation of dimerization free energy (see below) between the experimental values and the values obtained using the new Martini 3 in contrast to the old Martini 2 version [62]. This study also explained that the CG representation allows the prediction of the native-like structure of the TM dimer and is comparable with the available experimental data on the configuration of TMD helix dimers. Therefore, application of all these in silico methods to TMD dimerization gives not only insight into the spatial organization of a TMD dimer but also provides opportunities to explore its dynamics and those of the peptide surrounding lipids, waters and ions. In order to capture the details of the interactions, investigators often convert the CG structures to all-atom representations and allow an equilibration from 50 to several hundreds of ns [e.g., [63], but see [64] and below].

### 2.3. Free Energy and Mechanism of TM Association

Ideally, if configurational space is sufficiently sampled in either CG or in AA simulations, a free energy landscape is derivable and provides a picture of valleys and crescents to estimate the probability/if not the frequency of transitions between different configurational/conformational states using Markov modelling. Overall, the free energy of dimerization is another validation of the prediction of the structure of TM oligomers [20]. First, it allows the selection of the most favorable configuration from the set of structural models. Second, different dimers can be compared by their free energy value and can be arranged according to their strength of dimerization. Eventually, the comparison of TM dimers having similar sequences (considering the wild-type TM protein and their mutants) can display crucial information about the functional role of interface residues in dimerization. As mentioned, especially consideration of residues showing different protonation states are important in order to obtain crucial insights into the effects of pH in dimerization. However, statically assigned charge states of residues do not give a clear picture of that ionization states, as Asp/Glu charged in solution, for example may be buried in the membrane when neutralized by protonation, with their pKa having been significantly shifted. Such residues are used in some peptides to make them sensitive to pH changes and to force a particular alignment of TM helices, as contacts with another polar sidechain group, rather than with an aliphatic group are favored. Recently, constant pH MD methods have been developed to allow pKi’s and ionization states to shift [65].

Rather than running exhaustive sampling calculations of configurational space, which would be very computationally expensive in an explicit solvent and membrane environment, the free energy of association can be calculated through the potential of mean force (PMF) from a set of umbrella sampling simulations, which can be run in AA or CG representation. PMF provides a complete description of thermodynamic properties along a selected number of degrees of freedom, that is, a reaction coordinate- typically a distance between the helical TMD peptides. [66]. This technique has been used in the past to study the TMD association of Glycophorin A (GpA) and several of its mutants [67]. The CG representation of the TM peptides and the membrane are also helpful in estimating the free energy of TM dimerization even in the absence of an experimentally solved dimeric structure. Such an approach was applied for the determination of association energy and its difference for several TMD helix dimers including GpA and EphA1 [56,58].

Apart from the usual caveats concerning the completeness of conformational/configurational sampling and the accuracy of the potential energy function (most recently Best and colleagues showed a shortcoming of CHARMM36 in lipid bilayers [64], which can be corrected, however, while CG simulations may be more accurate), there are other issues. There is the likely adjustment of some charged residues (see constant pH dynamics above) but also that polarizable potential functions are likely more accurate than point (partial) charges. Another technical point concerns the CG simulations with Martini 3 which, like its predecessor, needs to use restraints to maintain regular secondary structures. Thus, it is difficult to reliably sample helix-coil (un-)folding transitions at the ends of helices for example.

Generally, the accuracy of all the above computational methods is validated by comparison of the structures obtained with those of experimentally determined TMD complexes, by NMR either in several membrane mimics, like detergent micelles or in lipid bicelles/nanodiscs. While the membrane mimicking model systems are still a challenge for some of the experimental structure determinations, the CG and AA-MD simulations are nowadays able to relatively quickly sample different membrane compositions, solution conditions and mutant forms of the TMDs, not easily accessible experimentally. This makes the computational techniques very powerful, exploring several possibilities of TM association which are energetically favorable and are often functionally important.

## 3. Association of Inhibitor/Activator Peptides as an Avenue to Integrate TMD Behavior with Whole-Length Eph Receptor Function: Fluorescence-Based Experiments

When computational methods are used to study the association of TM helices in model systems, of course missing from the calculations are the effects of the extracellular and intracellular domains of the receptor protein. Also, the interactions between the extracellular region (LBD-LBD and LBD-FN domains) are important for receptor oligomerization and clustering [68]. Yet it’s known that in some cases those surrounding domains interact at least transiently with the membrane [69]. This has been suggested by CG simulations of the membrane proximal fibronectin domain of EphA2 [70] as well as its kinase domain [63]. In some systems, such interactions can have a profound effect on the structures of the TMDs which are populated and the question of how preferences for a certain state of the TMD synergize, oppose or are neutral with respect to the remainder of the protein is an urgent one to resolve for EphA2. (CG simulations with larger regions of the EphA1 and −A2 receptors surrounding their TMDs are in progress in the Buck lab, also of the full-length proteins). Experimentally, there are several avenues to characterize the effect of the TMD on the functional, if not structural behavior of the whole length receptor. One is the study of the TMD/whole length receptor with inhibitor/activator TMD-like peptides, the other one is the characterization of the effects of mutations in the TMD. Both approaches are best utilized in conjunction with fluorescence-based techniques which detect either the proximity of fluorescent proteins or labels (FRET—Förster resonance energy transfer) or their correlated movement in solution or in cells (PIE-FCCS; pulsed interleaved excitation-fluorescence cross-correlation spectroscopy). Because no studies have yet been done on TMD mutants for Eph receptors, we refer the reader to excellent work done using these fluorescence techniques on other TMD systems, esp. EGFR and to several reviews [71,72,73,74].

**Figure 4:**
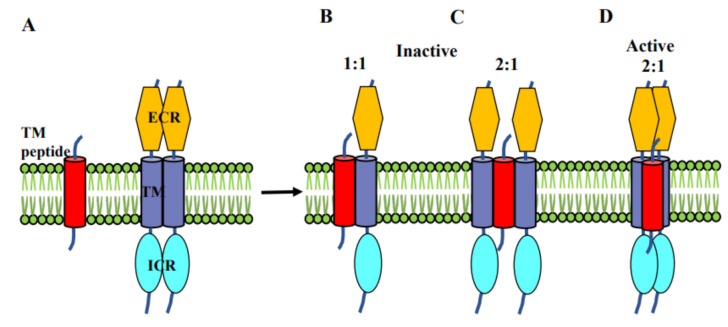
Schematic representing the association of inhibitor/activator peptides with Eph receptor dimer showing the involvement of TM domains (**A**). (**B**,**C**) Inhibitor peptide breaks the receptor dimerization and makes either 1:1 or 2:1 (receptor: peptide) interaction and thereby inactivating the receptor whereas (**D**) the activator peptide associate with the receptor dimer in 2:1 (receptor: peptide) fashion without breaking the TM association.

Very recently, several studies with peptides consisting of only the TMD of the receptors have provided crucial insights into the receptor activation and function (Figure 4) [74]. These TMD peptides interact with the target receptor especially the TMD and thereby may inhibit the receptor dimerization. However, TMD peptides targeting other receptors, including EphA2 have been shown to induce receptor oligomerization. Therefore, a detailed structural and dynamics characterization of the interaction between these TMD peptides and their target receptors may also be helpful in providing insights into receptor activation mechanisms. Nguyen and colleagues recently designed the acidity-triggered rational membrane (ATRAM) peptide [75]. ATRAM is a highly soluble synthetic peptide that is capable of pH-dependent interaction with lipid membranes: at neutral pH, ATRAM binds to the membrane surface, while a decrease in pH triggers insertion into the lipid bilayer as a TM helix. Similarly, the recently designed TYPE7 peptide is also highly soluble in the aqueous solution that inserts into cellular membranes at slightly acidic pH. The TM state of TYPE7 interacts with EphA2 and induces receptor oligomerization and phosphorylation [27,28]. Using CG MD simulation our laboratory has helped to suggest a mechanism by which the TYPE7 peptide stabilizes the active configuration of the helix dimer by forming a 2:1 (EphA2 TMs: peptide) trimer complex and thereby promoting EphA2 oligomerization. As an aside it is interesting to note that such bivalent interactions are also the mechanism by which a designed peptide promotes receptor oligomerization and activation by bridging the extracellular ligand binding domain between two receptors [76].

In other systems, for example in plexin-A1 interacting with its coreceptor neuropilin, a neuropilin TMD-like peptide was found to inhibit the function of the plexin receptor [77]. Generally, one can envisage two mechanisms for this. The TMD-like inhibitor peptide may stabilize a monomeric state of the receptor, as shown in Figure 4B. However, our preliminary data suggest that the plexin is not monomerized, rather the TMD is added to the helix dimer, stabilizing the inactive state (Figure 4C). Conversely, activation by a peptide likely, such as computationally modeled for TYPE7 involves an interaction of the TMD-like peptide with both EphA2 TM helices of the full-length receptor (Figure 4D). This is appropriate for RTK activation as it involves the coming together of receptors to form dimer or high order associations which bring the ICR kinase domains together to allow their cross-phosphorylation and activation.

## 4. Concluding Summary and Perspectives

We are still in the process of understanding the structural components involved in Eph receptor dimerization/oligomerization and also Eph-ephrin interaction, possibly *in cis* [77] as well as *in trans*, all features which contribute to-, if not comprise the regulatory mechanism of Eph receptor signaling. This review discussed studies on the transmembrane domain (TMD) of EphA1 and -A2 which have been studied so far and provided an overview of the experimental and computational techniques used. From the reported results it is clear that the TMD has a significant role to play in signal transduction across the plasma membrane. Particularly understanding the relationship between how TM helix dimerization motifs predispose the TMD to populate particular configurational states and the associated level of dimerization and the activity of the whole length receptor is of key importance for its further characterization. This importance is suggested by the finding that a TMD-like peptide can critically influence the TMD configurational structure and whole receptor function [64]. The next steps in this challenging work will be to test the TMD dimerization/oligomerization motifs by mutagenesis in conjunction with NMR on receptor fragments which contain the TMD but also membrane proximal domains and to integrate the behavior of the TMD with these regions and eventually with the function of the full-length receptor. The community of researchers studying single-pass transmembrane receptors is eagerly awaiting a medium to high resolution structure of a full-length receptor, most likely coming from cryo-EM, once the issues with models for the lipid bilayer have been solved. Such a model not only needs to surround the TMD but may also need to interact with membrane proximal and possibly distal domains. It could be a while before large enough lipid bilayer model systems are developed to allow such extensive protein-membrane interactions, and also the larger scale clustering which is observed upon Eph receptor activation. Cryo-electron tomography, the observation of Eph receptors at high enough resolution at native cell membranes would be an ultimate feat, but supported lipid bilayer systems, which allow sufficient space for the intracellular region could be an alternative. Early steps for producing and purifying full-length EphA2 were made in the Nikolov lab [78]. It may be worth remembering that many instances of biological function have been organized in a hierarchical manner and we should have some confidence that the reductionist approach of studying protein fragments. Fragments such as the TMD have already provided- and are likely to yield more critical insights including avenues for therapeutic approaches in the future.

## Data Availability

Not applicable.
